# Analyzing inherent biases in SARS-CoV-2 PCR and serological epidemiologic metrics

**DOI:** 10.1186/s12879-022-07425-z

**Published:** 2022-05-13

**Authors:** Monia Makhoul, Farah Abou-Hijleh, Shaheen Seedat, Ghina R. Mumtaz, Hiam Chemaitelly, Houssein Ayoub, Laith J. Abu-Raddad

**Affiliations:** 1 Infectious Disease Epidemiology Group, Weill Cornell Medicine-Qatar, Cornell University, Doha, Qatar; 2 World Health Organization Collaborating Centre for Disease Epidemiology Analytics On HIV/AIDS, Sexually Transmitted Infections, and Viral Hepatitis, Weill Cornell Medicine-Qatar, Qatar-Foundation-Education City, Cornell University, P.O. Box 24144, Doha, Qatar; 3 Department of Population Health Sciences, Weill Cornell Medicine, Cornell University, New York, NY USA; 4 Department of Public Health, College of Health Sciences, Academic Quality Affairs Office, QU Health, Qatar University, Doha, Qatar; 5 Department of Epidemiology and Population Health, American University of Beirut, Beirut, Lebanon; 6 Mathematics Program, Department of Mathematics, Statistics and Physics, College of Arts and Sciences, Qatar University, Doha, Qatar

**Keywords:** SARS-CoV-2, PCR positivity, Antibody detection, Bias, Incidence, Prevalence, Seroprevalence, Epidemic

## Abstract

**Background:**

Prospective observational data show that infected persons with the severe acute respiratory syndrome coronavirus 2 (SARS-CoV-2) remain polymerase chain reaction (PCR) positive for a prolonged duration, and that detectable antibodies develop slowly with time. We aimed to analyze how these effects can bias key epidemiological metrics used to track and monitor SARS-CoV-2 epidemics.

**Methods:**

An age-structured mathematical model was constructed to simulate progression of SARS-CoV-2 epidemics in populations. PCR testing to diagnose infection and cross-sectional surveys to measure seroprevalence were also simulated. Analyses were conducted on simulated outcomes assuming a natural epidemic time course and an epidemic in presence of interventions.

**Results:**

The prolonged PCR positivity biased the epidemiological measures. There was a lag of 10 days between the *true* epidemic peak and the *actually-observed* peak. Prior to epidemic peak, PCR positivity rate was twofold higher than that based only on current active infection, and half of those tested positive by PCR were in the prolonged PCR positivity stage after infection clearance. Post epidemic peak, PCR positivity rate poorly predicted true trend in active infection. Meanwhile, the prolonged PCR positivity did not appreciably bias estimation of the basic reproduction number *R*_0_. The time delay in development of detectable antibodies biased measured seroprevalence. The *actually-observed* seroprevalence substantially underestimated *true* prevalence of ever infection, with the underestimation being most pronounced around epidemic peak.

**Conclusions:**

Caution is warranted in interpreting PCR and serological testing data, and any drawn inferences need to factor the effects of the investigated biases for an accurate assessment of epidemic dynamics.

**Supplementary Information:**

The online version contains supplementary material available at 10.1186/s12879-022-07425-z.

## Background

The severe acute respiratory syndrome coronavirus 2 (SARS-CoV-2) emerged in late 2019 [1] and resulted in a pandemic [2]. As the number of laboratory-confirmed cases and coronavirus disease 2019 (COVID-19) related deaths continue to rise [2], this virus will persist as a global public health concern.

At present, the main diagnostic modality for SARS-CoV-2 is the polymerase chain reaction (PCR) test, which is typically performed on either an upper or lower respiratory tract sample. Current understanding of the infection course suggests a latent phase of few days, followed by an infectious phase also of few days before recovery [1, 3–6]. Individuals infected with the virus test positive by PCR if tested during these first two stages, but also can test positive during the recovery stage for 2–4 weeks, reflecting genetic remnants of the virus [7, 8]. The latter duration defines the *prolonged PCR positivity* duration following end of infectiousness.

Individuals recovered from this infection also typically do not develop *detectable* IgG antibodies immediately, but 2–4 weeks thereafter [7, 8]. The latter duration defines the *pre-antibody positivity* duration following end of infectiousness.

The presence of the prolonged PCR positivity duration and the pre-antibody positivity duration can complicate the epidemiological inferences drawn from population-based testing by PCR and serological assays. Using mathematical modeling simulations, the aim of the present study is to analyze how these durations can bias the key epidemiological metrics that are used to track and monitor SARS-CoV-2 epidemics, for the purpose of improving interpretation of PCR and serological testing data and our understanding of local epidemics, but also for better management of the adverse implications of the social and physical distancing restrictions.

## Methods

### Mathematical model

An age-structured mathematical model was developed to simulate SARS-CoV-2 transmission dynamics in a given generic population (Additional file 1: Fig. S1), as informed by recent modeling studies [9–13]. The model was structured factoring current understanding of SARS-CoV-2 epidemiology, and stratifies the population into compartments according to age group, infection status, infection stage, and disease stage. Following a latency period, infected individuals progress to either asymptomatic/mild infection followed by recovery, or they progress to severe or critical infection. Severe or critical infection progresses to severe or critical disease, respectively, prior to recovery, but critical disease cases have an additional risk for COVID-19 mortality.

The model further includes three tracking population compartments for the prolonged PCR positivity, pre-antibody positivity, and antibody positivity. Informed by empirical evidence [7, 8], it was assumed that infected individuals remain in the prolonged PCR positivity stage for 3 weeks on average and in the pre-antibody positivity stage also for 3 weeks on average. Some of the analyses below explored the impact of other values for these durations.

Description of the model structure, equations, and parameters are in the Additional file 1. All analyses were conducted on the MATLAB R2019a platform.

### Analysis scenarios

Two types of SARS-CoV-2 epidemics were simulated in this generic population: one assuming a basic reproduction number (*R*_*0*_) of 3.0, reflecting the natural course of the epidemic in *absence* of any social or physical distancing interventions [14, 15], and one assuming an *R*_*0*_ of 1.6, reflecting an epidemic in *presence* of these interventions, such as that of Qatar where *R*_*0*_ was estimated at about 1.6 [10].

Random PCR testing was simulated on this population through Monte Carlo sampling. Trend in PCR positive diagnoses was generated assuming first that individuals are PCR positive *only* during infection latency and infectiousness (that is during *only* active infection), and then assuming that individuals are PCR positive during infection latency, infectiousness, *and* the prolonged PCR positivity following end of infectiousness. These two simulated trends represent thus the *true* active infection presence in the population and the *actually-observed* presence through PCR testing, respectively.

Repeated *daily* cross-sectional surveys to measure antibody prevalence (seroprevalence) were also simulated on this population by Monte Carlo sampling a random sample every day. The trend in seroprevalence was generated assuming that individuals develop detectable antibodies *immediately* following onset of infection (that is detectable antibodies reflect actual infection *once* the infection occurs), and then assuming that individuals develop detectable antibodies *only* after passing through the stage of pre-antibody positivity following end of infectiousness. Once antibodies develop, it was assumed that they would persist for a long duration, beyond the simulation timeframe. These two simulated trends represent thus the *true* prevalence of ever infection in the population and the *actually-observed* seroprevalence as measured using serological assays, respectively.

## Results

**Fig. 1:**
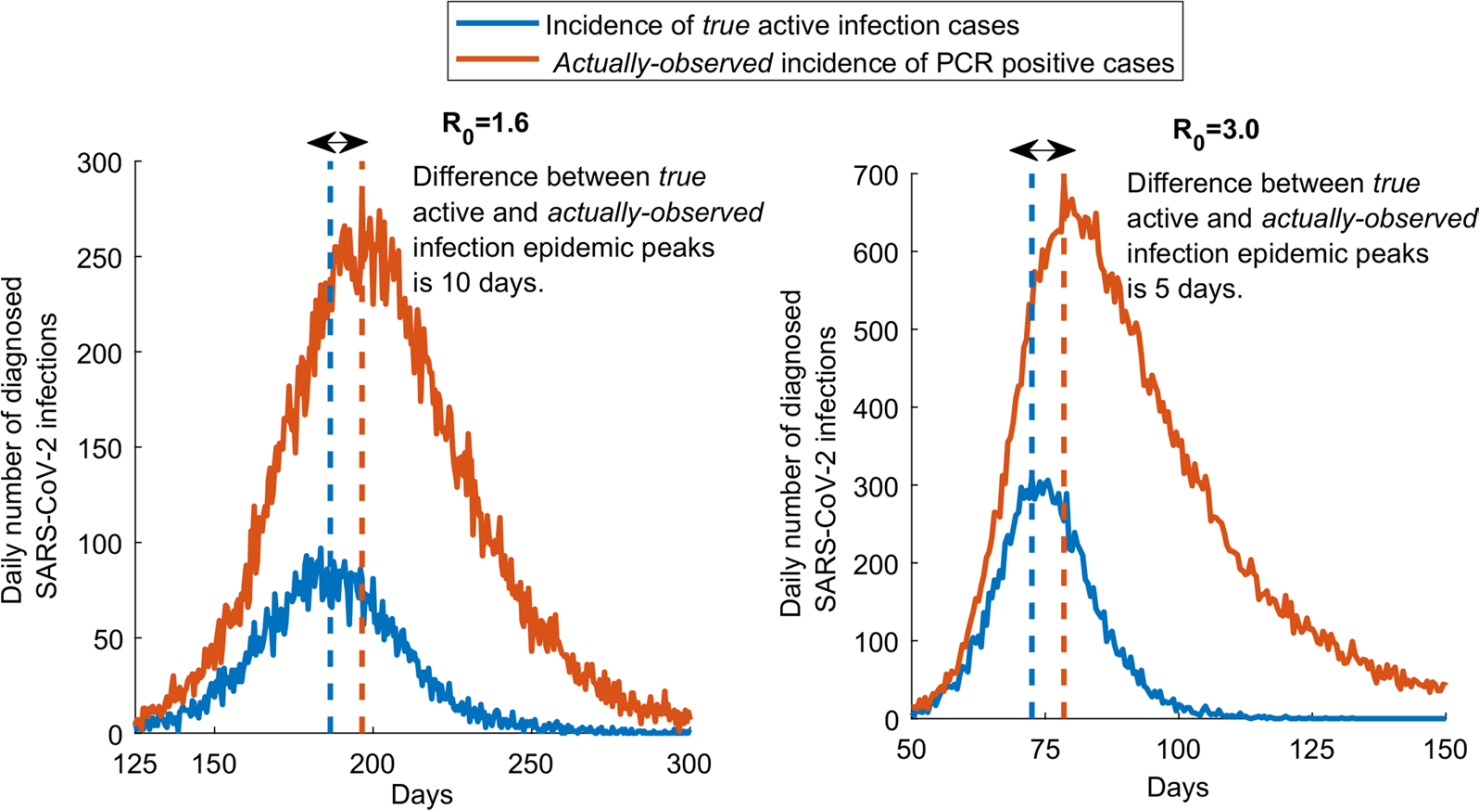
Effect of the prolonged PCR positivity on the observed trend of diagnosed cases. Daily number of new diagnosed cases of *true* active infection versus the *actually-observed* diagnosed cases in presence of the prolonged PCR positivity. The prolonged PCR positivity is assumed to last on average for three weeks after end of infectiousness [7, 8]. Two scenarios are presented, one for an *R*_0_ of 1.6 (an epidemic in presence of social and physical distancing interventions) and an *R*_0_ of 3.0 (natural course of the epidemic in absence of any social or physical distancing interventions)

Figure 1 shows the simulated daily number of PCR-positive diagnosed cases in the scenario that PCR positivity measures *true* active infection presence in the population compared to the *actually-observed* scenario in presence of the prolonged PCR positivity. There is a lag of 10 days between the *true* peak in infection incidence and the *actually-observed* peak in infection incidence when *R*_0_ is 1.6, and a lag of 5 days when *R*_0_ is 3.0. Moreover, the scenario incorporating the prolonged PCR positivity results in more cases being diagnosed than the scenario in which infected individuals are PCR positive only during active infection.

**Fig. 2:**
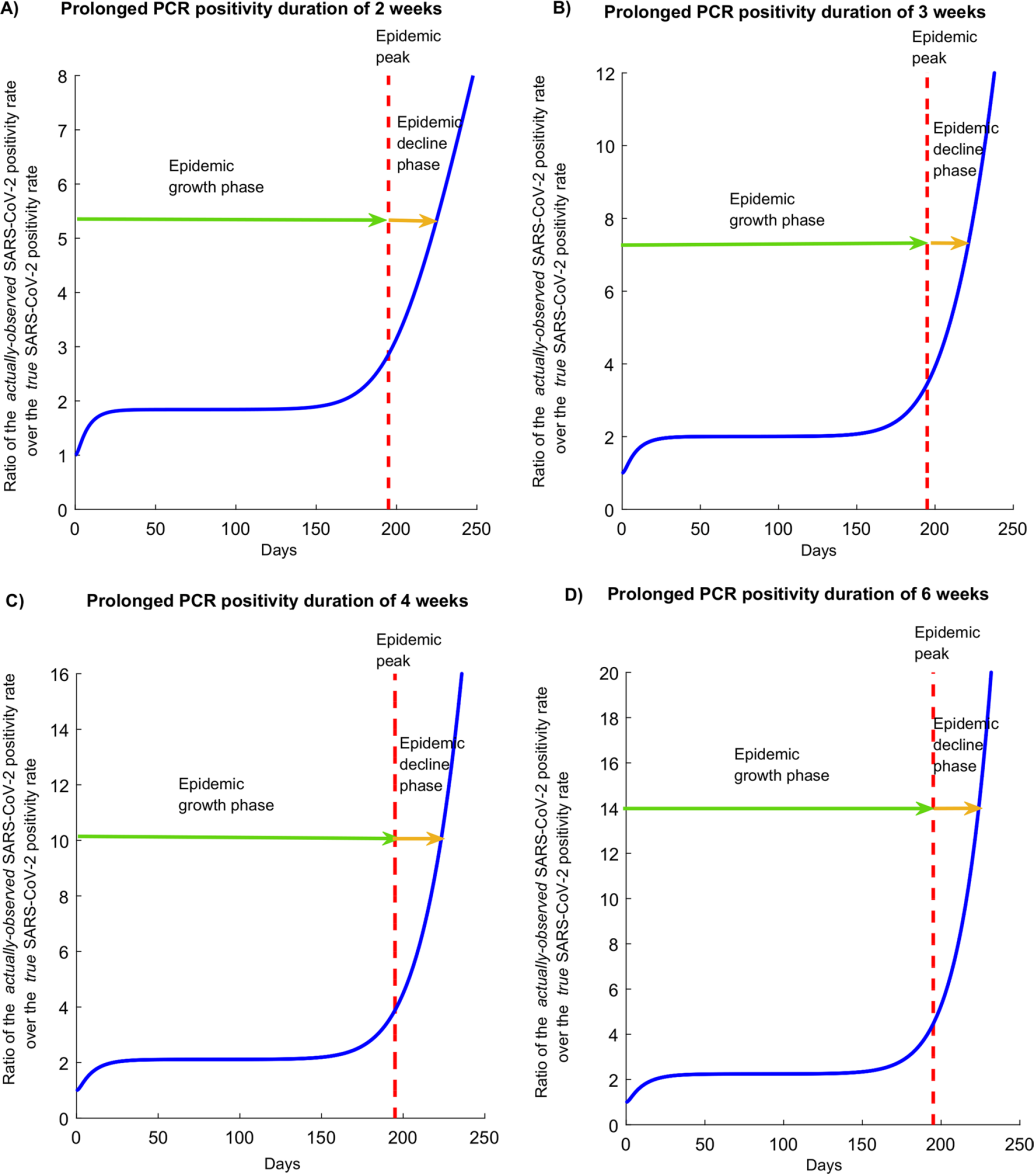
Effect of the prolonged PCR positivity on the observed SARS-CoV-2 positivity rate. Ratio of the proportion of tests that are PCR positive (“positivity rate”) in presence of the prolonged PCR positivity over the proportion of tests that are PCR positive assuming no prolonged PCR positivity. The prolonged PCR positivity is assumed to last on average for 2, 3, 4, and 6 weeks. In this epidemic simulation, *R*_0_ has a value of 1.6, that is an epidemic time course in presence of social and physical distancing interventions. The simulation for *R*_0_ of 3.0, that is for the natural course of the epidemic in absence of any social or physical distancing interventions, is found in Figure S2

Figure 2 shows the ratio of the proportion of tests that are PCR positive (“positivity rate”) in presence of the prolonged PCR positivity divided by the proportion of tests that are PCR positive assuming no prolonged PCR positivity. This ratio is shown assuming a prolonged PCR positivity duration of 2, 3, 4, or 6 weeks. Prior to the epidemic peak, the proportion of tests that are PCR positive in presence of the prolonged PCR positivity is twofold higher than that assuming no prolonged PCR positivity. Meanwhile, after the epidemic peak, the ratio of the two proportions steadily increases and is higher the longer is the prolonged PCR positivity—that is more and more of the infections are diagnosed not during active infection, but during the prolonged PCR positivity stage. These results were generated assuming an *R*_0_ of 1.6, and the results assuming an *R*_0_ of 3.0 show the same pattern (Additional file 1: Fig. S2).

**Fig. 3:**
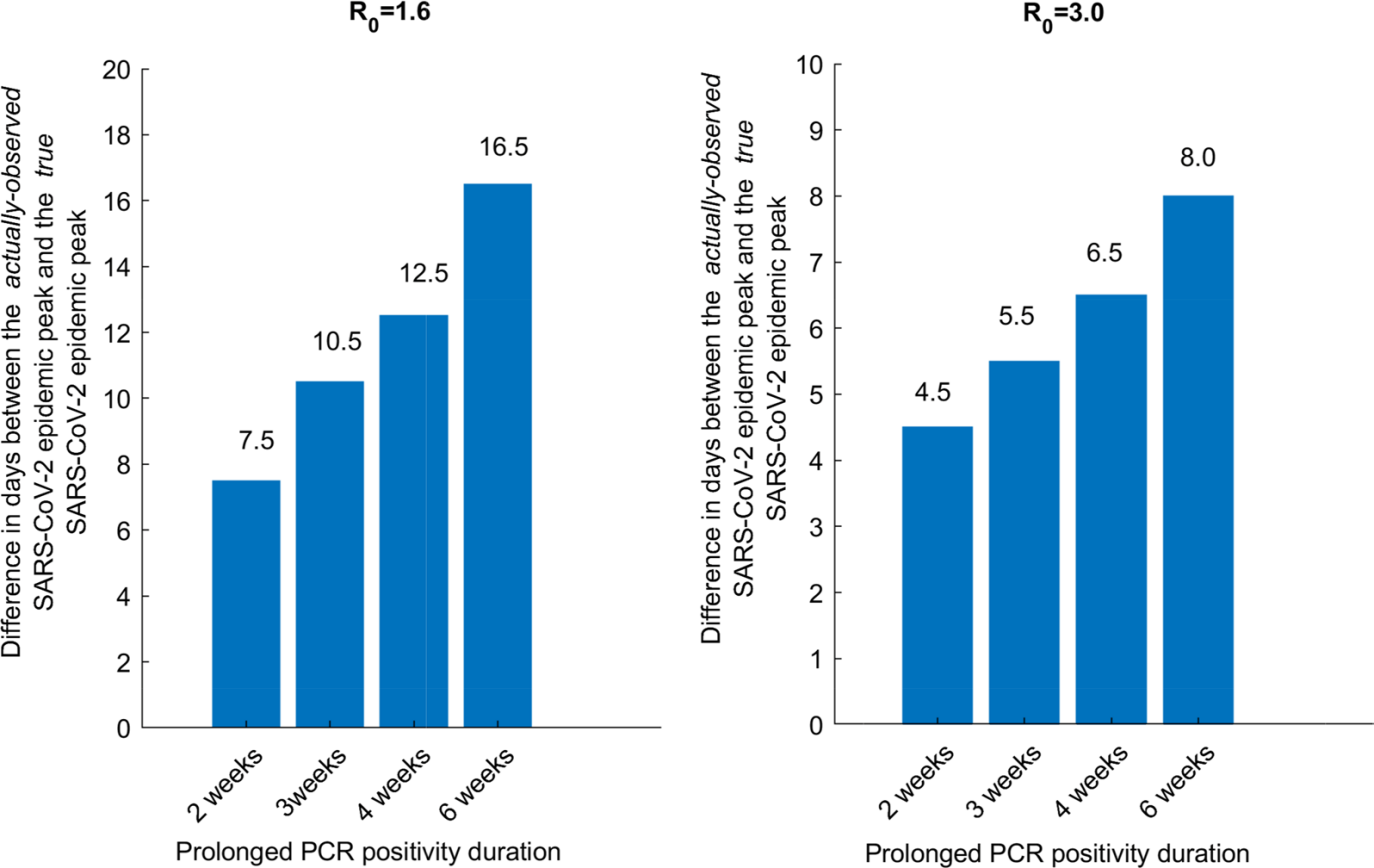
Effect of the prolonged PCR positivity on the observed SARS-CoV-2 epidemic peak. Time difference between the *actually-observed* epidemic peak in presence of the prolonged PCR positivity and the *true* epidemic peak based on true incidence of active infection in the population. The prolonged PCR positivity is assumed to last on average for 2, 3, 4, and 6 weeks. Two scenarios are presented, one for an *R*_0_ of 1.6 (an epidemic in presence of social and physical distancing interventions) and an *R*_0_ of 3.0 (natural course of the epidemic in absence of any social or physical distancing interventions)

Figure 3 presents the difference in days between the epidemic peak as measured in presence of the prolonged PCR positivity and the epidemic peak based on true incidence of active infection in the population, assuming a prolonged PCR positivity duration of 2, 3, 4, or 6 weeks. The delay between the *true* epidemic peak and the *observed* epidemic peak increased as the duration of prolonged PCR positivity increased. This delay ranged from 7.5 days up to 16.5 days at an *R*_0_ of 1.6, and from 4.5 days up to 8.0 days at an *R*_0_ of 3.0.

**Fig. 4:**
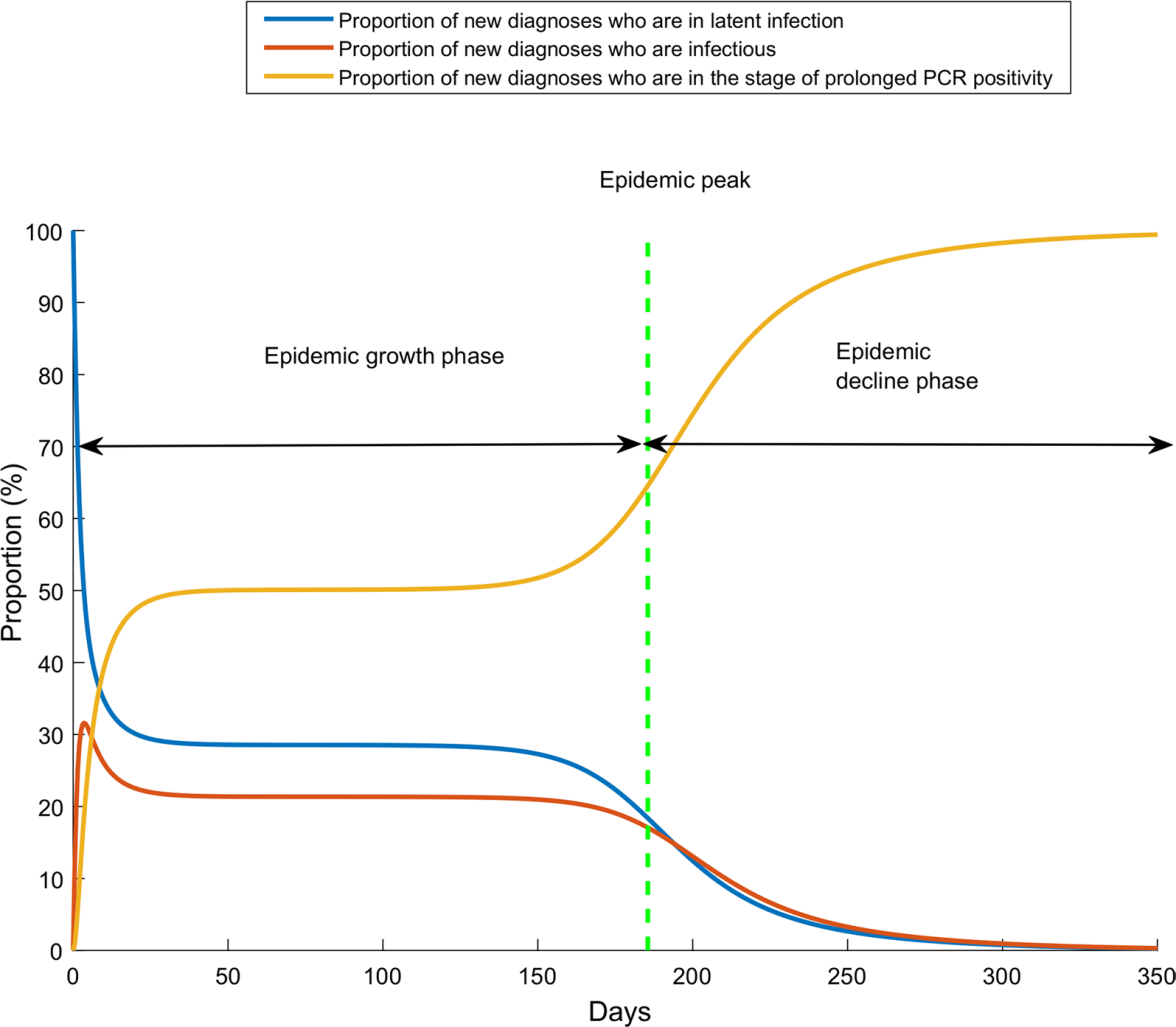
Effect of the prolonged PCR positivity on distribution of those latently infected, infectious, and post-infectious. Proportion of new diagnoses who are in latent infection, stage of infectiousness, or stage of prolonged PCR positivity. The prolonged PCR positivity is assumed to last on average for three weeks after end of infectiousness [7, 8]. In this epidemic simulation, *R*_0_ has a value of 1.6, that is an epidemic time course in presence of social and physical distancing interventions. The simulation for *R*_0_ of 3.0, that is for the natural course of the epidemic in absence of any social or physical distancing interventions, is found in Additional file 1: Fig. S3

Figure 4 and Additional file 1: Fig. S3 illustrate the change throughout the epidemic in the proportion of those who test positive by PCR and are latently infected, infectious, or post-infectious (that is in the prolonged PCR positivity stage) for $R_{0} = 1.6$ and $R_{0} = 3.0$, respectively. For $R_{0} = 1.6$, prior to the epidemic peak, approximately half of those who test positive by PCR are in the prolonged PCR positivity stage (that is already recovered from the infection). After the epidemic peak, this proportion rises steeply as the epidemic begins to decline. A similar pattern is seen for $R_{0} = 3.0$ (Additional file 1: Fig. S3).

Figure S4 of the Additional file 1 shows the estimated *R*_0_ as derived from the epidemic curve of diagnosed cases *in presence* and *in absence* of the prolonged PCR positivity. Two scenarios are presented, the first for an *R*_0_ of 1.6, and the second for an *R*_0_ of 3.0, and each factoring a prolonged PCR positivity duration of 2, 3, 4, or 6 weeks. The estimated *R*_0_ from the *actually-observed* diagnosed cases is always lower than that estimated from the *true* (active infection) diagnosed cases, but the difference is small, particularly so for the case of *R*_0_ of 1.6, and is not much affected by the duration of the prolonged PCR positivity.

**Fig. 5:**
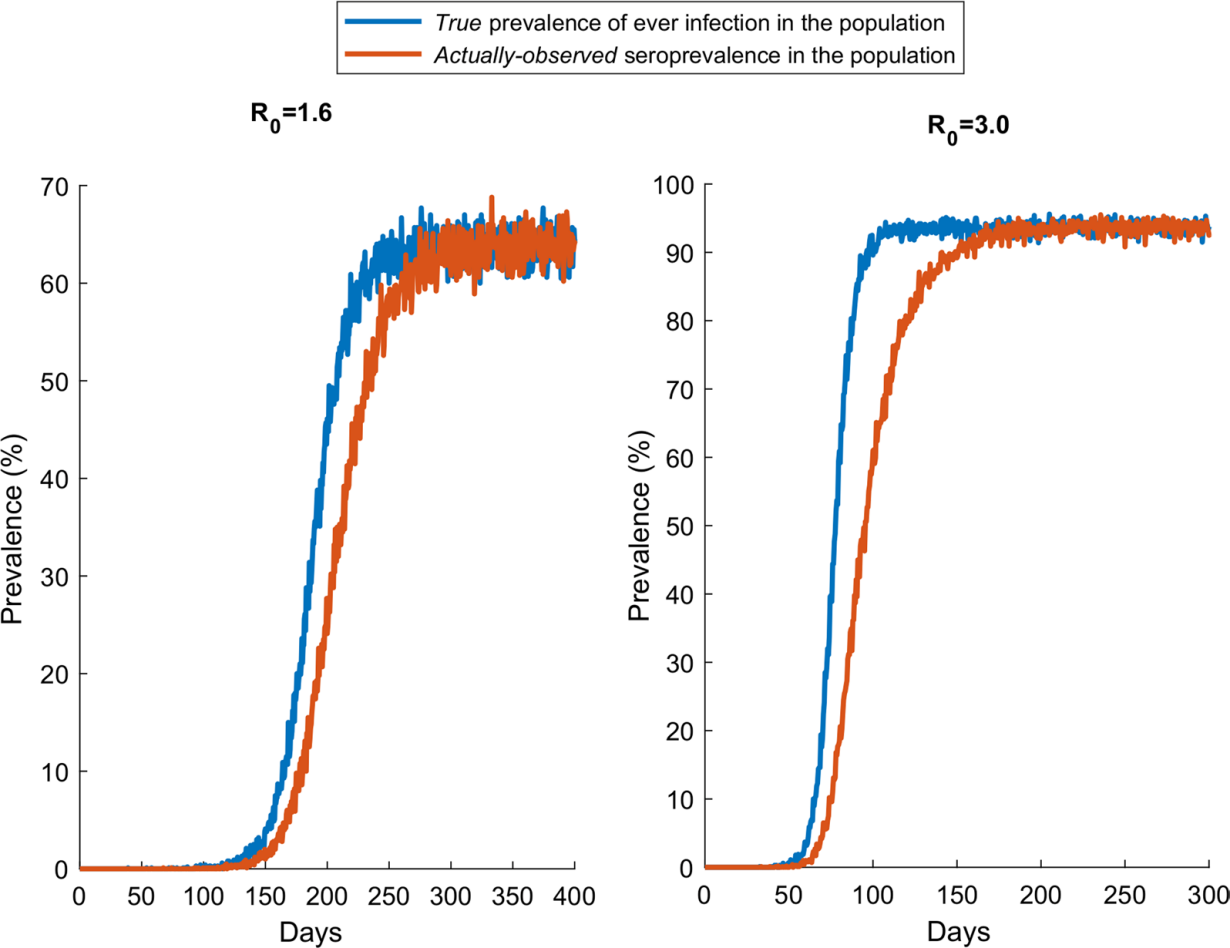
Effect of delay in development of detectable antibodies on observed seroprevalence in cross-sectional surveys. Trend in the *true* prevalence of ever infection in the population versus the *actually-observed* seroprevalence factoring the 3 weeks average delay in the development of detectable antibodies [7, 8]. Two scenarios are presented, one for an *R*_0_ of 1.6 (an epidemic in presence of social and physical distancing interventions) and an *R*_0_ of 3.0 (natural course of the epidemic in absence of any social or physical distancing interventions)

Figure 5 shows the trend in the *true* prevalence of ever infection in the population versus the *actually-observed* seroprevalence factoring the 3 weeks average delay in the development of detectable antibodies [7, 8]. Two scenarios are presented, the first for an *R*_0_ of 1.6 and the second for an *R*_0_ of 3.0. There is a time delay in the *actually-observed* seroprevalence reaching the *true* prevalence of ever infection in the population, and this delay varies with time reflecting the epidemic phase (particularly closeness to the epidemic peak) and the intensity of the epidemic (value of *R*_0_).

## Discussion

Management of an epidemic depends on availability of high quality real-time data in order to make the best decisions. Above results show that presence of the prolonged PCR positivity, one of the distinctive features of the SARS-CoV-2 infection reflecting the presence of genetic remnants of the virus in those who cleared their infection [7, 8], biases the epidemiological metrics and inferences drawn from the trend of PCR-positive diagnosed cases. While the prolonged PCR positivity allows more infections to be diagnosed (Fig. 1), it biases assessment of the epidemic phase. The *true* phase of the epidemic (epidemic peak and also epidemic growth or decline) occurs 1–2 weeks *before* the *actually-observed* phase of the epidemic (Figs. 1 and 3). This implies that the trend in PCR-positive diagnosed cases does not reflect the current status of the epidemic, but the status 1–2 weeks earlier. However, the prolonged PCR positivity does not appreciably bias the derivation of *R*_*0*_ from the actually-observed trend in diagnosed cases (Additional file 1: Fig. S4).

The prolonged PCR positivity also biases the testing positivity rate. The *actually-observed* positivity rate is twofold higher than the *true* positivity rate of active infection during the epidemic growth phase, and several folds higher during the epidemic decline phase (Fig. 2). As the epidemic declines, the value of the positivity rate in conveying the actual epidemic dynamics erodes steadily with time. Moreover, during the epidemic growth phase, as much as half of those who test positive by PCR are in the prolonged PCR positivity stage having already recovered from the infection. During the epidemic decline phase, increasingly most of those testing positive are found in the prolonged PCR positivity stage and not in active infection (Fig. 4). Remarkably, at all times, those newly diagnosed with the infection are likely to be found in a non-infectious stage.

Above results demonstrate that the documented time delay in development of detectable antibodies [7, 8] biases measures of seroprevalence that are derived from cross-sectional surveys of the population. At all times prior to end of the epidemic cycle, *actually-observed* seroprevalence underestimates *true* prevalence of ever infection in the population (Fig. 5). The difference between what is actually observed and what is true is most pronounced around the epidemic peak. This finding demonstrates that current seroprevalence studies can significantly underestimate actual infection exposure in the population at large; an important consideration given that communities are increasingly undertaking sero-surveys to understand better virus spread and to develop efficient plans for managing the hefty costs of the social and physical distancing restrictions on society and economy. This finding has been empirically-confirmed recently where it has been shown that seroprevalence substantially underestimated *true* ever-infection prevalence in a PCR and serological survey [16].

Empirical studies reported that PCR positivity rate for SARS-CoV-2 was more than 90% at 3–5 days after onset of symptoms, but decreased to less than 60% at 6 days, and to less than 50% at 14 days [17, 18]. Meanwhile, the median duration of antibody detection after onset of symptoms was 5 days [interquartile range (IQR): 3–6] for IgM and IgA and 14 days (IQR: 10–18) for IgG, with antibody positivity rate of 85.4%, 92.7%, and 77.9%, respectively [18]. The detection efficiency by IgM ELISA was higher than that of PCR after 5.5 days from onset of symptoms, and the positive detection rate was significantly increased (98.6%) when combining results of an IgM ELISA assay with those of PCR for each patient compared with using only the result of a single PCR test (51.9%) [18]. This evidence indicates that using the combined results of PCR and IgM, IgA and IgG antibody testing simultaneously can address some of the biases documented in the present study and improve the interpretation of epidemic dynamics.

This study has limitations. PCR testing was assumed random, but in reality this depends on the actual testing policy that is enforced in any setting. For instance, administering PCR testing to only those presenting with clinical symptoms will differentially bias detection towards those who acquired the infection within the last 5–10 days. The above results thus need to be complemented with further analysis for each specific setting to factor the actually-enforced testing policy. While the prolonged PCR positivity and the time delay in development of detectable antibodies are well-established in the literature [7, 8], it is still not sufficiently known how these are distributed in the population by age and sex, factors that may influence these effects and their impact on epidemiological measures. Epidemiological metrics can also be biased by other aspects of PCR and antibody testing, such as assay sensitivity and specificity, which are not investigated in the present study.

## Conclusions

The prolonged PCR positivity observed in SARS-CoV-2 infected persons and the time delay in development of detectable antibodies can bias key epidemiological metrics used to track and monitor SARS-CoV-2 epidemics leading to implications for managing the social and physical distancing restrictions. Caution is warranted in interpreting PCR and serological testing data, and any drawn inferences need to factor these biases for an accurate assessment of epidemic dynamics. These findings also suggest that analysis of PCR and serological testing data should not only be based on dichotomous outcomes (positive versus negative), but should also factor the quantitative measures of PCR and serological assays (such as PCR cycle threshold and antibody optical density values) to improve interpretation of these metrics.

## Supplementary Information

**Additional file 1.Table S1.** Definitions of population variables and symbols used in the model. **Table S2.** Model assumptions in terms of parameter values. **Figure S1.** Schematic diagram presenting the basic structure of the mathematical model for SARS-CoV-2 transmission dynamics with the prolonged PCR positivity and delayed antibody detection. **Figure S2.** Effect of the prolonged PCR positivity on the observed SARS-CoV-2 positivity rate. **Figure S3.** Effect of the prolonged PCR positivity on the observed distribution of those who are latently infected, infectious, and post-infectious. **Figure S4.** Effect of the prolonged PCR positivity on estimation of the basic reproduction number R_0_ using the *actually-observed* trend in diagnosed cases.

## Data Availability

The model’s MATLAB code can be found at the following URL: https://github.com/MouniaM/PCRCodesM.git.

## Abbreviations

SARS-CoV-2: Severe acute respiratory syndrome coronavirus 2
COVID-19: Coronavirus disease 2019
PCR: Polymerase chain reaction
