# Supplementary material for: Analyzing inherent biases in SARS-CoV-2 PCR and serological epidemiologic metrics
Source: BMC Infect Dis. 2022 May 13;22:458. doi: 10.1186/s12879-022-07425-z (PMC9100306; doi:10.1186/s12879-022-07425-z)
Supplement: Supplementary file 1 — Additional file 1.Table S1. Definitions of population variables and symbols used in the model. Table S2. Model assumptions in terms of parameter values. Figure S1. Schematic diagram presenting the basic structure of the mathematical model for SARS-CoV-2 transmission dynamics with the prolonged PCR positivity and delayed antibody detection. Figure S2. Effect of the prolonged PCR positivity on the observed SARS-CoV-2 positivity rate. Figure S3. Effect of the prolonged PCR positivity on the observed distribution of those who are latently infected, infectious, and post-infectious. Figure S4. Effect of the prolonged PCR positivity on estimation of the basic reproduction number R0 using the actually-observed trend in diagnosed cases. [file 12879_2022_7425_MOESM1_ESM.docx]

**Analyzing** **inherent biases in SARS-CoV-2 PCR and antibody epidemiologic metrics**

**Supplementary**

Monia Makhoul^1,2,3^, Farah Abou-Hijleh^4^, Shaheen Seedat^1,2,3^, Ghina R Mumtaz^5^, Hiam Chemaitelly^1,2^, Houssein Ayoub^6^, and Laith J. Abu-Raddad^1,2,3^

^1^Infectious Disease Epidemiology Group, Weill Cornell Medicine-Qatar, Cornell University, Doha, Qatar

^2^World Health Organization Collaborating Centre for Disease Epidemiology Analytics on HIV/AIDS, Sexually Transmitted Infections, and Viral Hepatitis, Weill Cornell Medicine-Qatar, Cornell University, Qatar-Foundation-Education City, Doha, Qatar

^3^Department of Population Health Sciences, Weill Cornell Medicine, Cornell University, New York, New York, USA

^4^Department of Public Health, College of Health Sciences, Academic Quality Affairs Office, QU Health, Qatar University, Doha, Qatar

*^5^Department of Epidemiology and Population Health, American University of Beirut, Beirut, Lebanon*

*^6^Department of Mathematics, Statistics, and Physics, College of Arts and Sciences, Qatar University, Doha, Qatar*

**Supplementary A**

1. **Mathematical model**

We constructed an age-structured deterministic compartmental mathematical model to quantify the impact of the prolonged polymerase chain reaction (PCR) positivity duration and delayed detection of antibodies on key epidemiological metrics for the severe acute respiratory syndrome coronavirus 2 (SARS-CoV-2) infection (Figure S1).

The model stratifies the population into compartments according to age group (0-9, 10-19, 20-29,…, ≥80 years), infection status (uninfected, infected), infection stage (mild, severe, critical), disease stage (severe, critical), and three other tracking compartments (prolonged PCR positivity, pre-antibody positivity, antibody positivity). The dynamics are described by age-specific sets of nonlinear ordinary differential equations, where each age group corresponds to a 10 years age band (0-9,10-19,…70-79) apart from the last age group incorporating those aged $\geq$80 years.

The following set of equations was used to describe the transmission dynamics for the first age group:

Population aged 0-9 years:

$$\frac{dS\left(1\right)}{dt}=-\left(\lambda\left(1\right)+\mu\left(1\right)+\xi\left(1\right)\right)S\left(1\right)$$

$$\frac{dE\left(1\right)}{dt}=\lambda\left(1\right)S\left(1\right)-\left(\delta+\mu\left(1\right)+\xi\left(1\right)\right)E\left(1\right)$$

$$\frac{dI_{M}\left(1\right)}{dt}=f_{M}\left(1\right)\delta E\left(1\right)-\left(\eta_{M}+\mu\left(1\right)+\xi\left(1\right)\right)I_{M}\left(1\right)$$

$$\frac{dI_{S}\left(1\right)}{dt}=f_{S}\left(1\right)\delta E\left(1\right)-\left(\eta_{DS}+\mu\left(1\right)+\xi\left(1\right)\right)I_{S}\left(1\right)$$

$$\frac{dI_{C}\left(1\right)}{dt}=f_{C}\left(1\right)\delta E\left(1\right)-\left(\eta_{DC}+\mu\left(1\right)+\xi\left(1\right)\right)I_{C}\left(1\right)$$

$$\frac{dD_{S}\left(1\right)}{dt}=\eta_{DS}I_{S}\left(1\right)-\left(\eta_{S}+\mu\left(1\right)+\xi\left(1\right)\right)D_{S}\left(1\right)$$

$$\frac{dD_{C}\left(1\right)}{dt}=\eta_{DC}I_{C}\left(1\right)-\left(\eta_{C}+\mu\left(1\right)+\xi\left(1\right)+\alpha\left(1\right)\right)D_{C}\left(1\right)$$

$$\frac{dR\left(1\right)}{dt}=\eta_{M}I_{M}\left(1\right)+\eta_{S}D_{S}\left(1\right)+\eta_{C}D_{C}\left(1\right)-\left(\mu\left(1\right)+\xi\left(1\right)\right)R\left(1\right)$$

The following set of equations was used to describe the transmission dynamics for all other age groups:

Population aged 10+ years:

$$\frac{dS\left(a\right)}{dt}=\xi\left(a-1\right)S\left(a-1\right)-\left(\lambda\left(a\right)+\mu\left(a\right)+\xi\left(a\right)\right)S\left(a\right)$$

$$\frac{dE\left(a\right)}{dt}=\xi\left(a-1\right)E\left(a-1\right)+\lambda\left(a\right)S\left(a\right)-\left(\delta+\mu\left(a\right)+\xi\left(a\right)\right)E\left(a\right)$$

$$\frac{dI_{M}\left(a\right)}{dt}=\xi\left(a-1\right)I_{M}\left(a-1\right)+f_{M}\left(a\right)\delta E\left(a\right)-\left(\eta_{M}+\mu\left(a\right)+\xi\left(a\right)\right)I_{M}\left(a\right)$$

$$\frac{dI_{S}\left(a\right)}{dt}=\xi\left(a-1\right)I_{S}\left(a-1\right)+f_{S}\left(a\right)\delta E\left(a\right)-\left(\eta_{DS}+\mu\left(a\right)+\xi\left(a\right)\right)I_{S}\left(a\right)$$

$$\frac{dI_{C}\left(a\right)}{dt}=\xi\left(a-1\right)I_{C}\left(a-1\right)+f_{C}\left(a\right)\delta E\left(a\right)-\left(\eta_{DC}+\mu\left(a\right)+\xi\left(a\right)\right)I_{C}\left(a\right)$$

$$\frac{dD_{S}\left(a\right)}{dt}=\xi\left(a-1\right)D_{S}\left(a-1\right)+\eta_{DS}I_{S}\left(a\right)-\left(\eta_{S}+\mu\left(a\right)+\xi\left(a\right)\right)D_{S}\left(a\right)$$

$$\frac{dD_{C}\left(a\right)}{dt}=\xi\left(a-1\right)D_{C}\left(a-1\right)+\eta_{DC}I_{C}\left(a\right)-\left(\eta_{C}+\mu\left(a\right)+\xi\left(a\right)+\alpha\left(a\right)\right)D_{C}\left(a\right)$$

$$\frac{dR\left(a\right)}{dt}=\xi\left(a-1\right)R\left(a-1\right)+\eta_{M}I_{M}\left(a\right)+\eta_{S}D\left(a\right)_{S}+\eta_{C}D_{C}\left(a\right)-\left(\mu\left(a\right)+\xi\left(a\right)\right)R\left(a\right)$$

The following equations were used to track the individuals who are PCR positive after the period of infectiousness (prolonged PCR positivity), individuals who cleared their infection but have not yet developed detectable antibodies (pre-antibody positivity), and individuals with detectable antibodies (antibody positivity):

Population aged 0-9 years:

$$\frac{dY_{PCR}\left(1\right)}{dt}=\eta_{M}I_{M}\left(1\right)+\eta_{DS}I_{S}\left(1\right)+\eta_{DC}I_{C}\left(1\right)-\left(\mu\left(1\right)+\vartheta_{Y_{PCR}}\left(1\right)+\xi\left(1\right)\right)Y_{PCR}\left(1\right)-\alpha\left(1\right)D_{C}\left(1\right)$$

$$\frac{dX_{IgG}\left(1\right)}{dt}=\eta_{M}I_{M}\left(1\right)+\eta_{DS}I_{S}\left(1\right)+\eta_{DC}I_{C}\left(1\right)-\left(\mu\left(1\right)+\vartheta_{X_{IgG}}\left(1\right)+\xi\left(1\right)\right)X_{IgG}\left(1\right)-\alpha\left(1\right)D_{C}\left(1\right)$$

$$\frac{dZ_{IgG}\left(1\right)}{dt}=\vartheta_{X_{IgG}}\left(1\right)X_{IgG}\left(1\right)-\left(\mu\left(1\right)+\xi\left(1\right)\right)Z_{IgG}\left(1\right)$$

Population aged 10+ years:

$$\begin{matrix} \frac{dY_{PCR}\left(a\right)}{dt}=\xi\left(a-1\right)Y_{PCR}\left(a-1\right)+\eta_{M}I_{M}\left(a\right)+\eta_{DS}I_{S}\left(a\right)+\eta_{DC}I_{C}\left(a\right)-\left(\mu\left(a\right)+\vartheta_{Y_{PCR}}\left(a\right)+\xi\left(a\right)\right)Y_{PCR}\left(a\right) \\ -\alpha\left(a\right)D_{C}\left(a\right) \end{matrix}$$

$$\begin{matrix} \frac{dX_{IgG}\left(a\right)}{dt}=\xi\left(a-1\right)X_{IgG}\left(a-1\right)+\eta_{M}I_{M}\left(a\right)+\eta_{DS}I_{S}\left(a\right)+\eta_{DC}I_{C}\left(a\right)-\left(\mu\left(a\right)+\vartheta_{X_{IgG}}\left(a\right)+\xi\left(a\right)\right)X_{IgG}\left(a\right) \\ -\alpha\left(a\right)D_{C}\left(a\right) \end{matrix}$$

$$\frac{dZ_{IgG}\left(a\right)}{dt}=\xi\left(a-1\right)Z_{IgG}\left(a-1\right)+\vartheta_{X_{IgG}}\left(a\right)X_{IgG}\left(a\right)-\left(\mu\left(a\right)+\xi\left(a\right)\right)Z_{IgG}\left(a\right)$$

The definitions of population variables and symbols used in the equations are listed in Table S1.

$$\lambda\left(a\right)=\beta\sigma\left(a\right)\sum_{a'=1}^{n_{age}} H_{a,a'}\frac{I_{M}(a')+I_{S}(a')+I_{C}(a')}{\left[S(a')+E(a')+I_{M}(a')+I_{S}(a')+I_{C}(a')+D_{S}(a')+D_{C}(a')+R(a')\right]}$$

Whereby $\beta$is the rate of infectious contacts and $\sigma(a)$ defines the susceptibility to infection across age groups.

The mixing among the different age groups is dictated by the mixing matrix $H_{a,a'}$. This matrix provides the probability that an individual in the $a$ age group will mix with an individual in the $a'$ age group. The mixing matrix is given by

$$H_{a,a'}=e_{Age}\delta_{a,a'}+(1-e_{Age})\frac{\left[S(a')+E(a')+I_{M}(a')+I_{S}(a')+I_{C}(a')+D_{S}(a')+D_{C}(a')+R(a')\right]}{\sum_{b=1}^{n_{age}} \left[S(b)+E(b)+I_{M}(b)+I_{S}(b)+I_{C}(b)+D_{S}(b)+D_{C}(b)+R(b)\right]}$$

Here, $\delta_{a,a'}$ is the identity matrix. $e_{Age}\in\left[0,1\right]$ measures the degree of assortativeness in the mixing. At the extreme $e_{Age}=0$, the mixing is fully proportional. Meanwhile, at the other extreme, $e_{Age}=1$, the mixing is fully assortative, that is individuals mix only with members in their own age group.

1. **Parameter values**

The model parameters were selected based on current empirical data for SARS-CoV-2 natural history and epidemiology, and as informed by a recent mathematical modeling application for Qatar [1, 2]. The parameter values are listed in Table S2.

1. **The basic reproduction number** $R_{0}$

Informed by Heffernan *et al.* [3], the basic reproduction number ($R_{0}$) is given as the weighted average of $R_{0}$ in each age group:

$$R_{0}=\sum_{a=1}^{n_{age}} w(a)\beta\sigma\left(a\right)\sum_{a'=1}^{n_{age}} H_{a,a'}\left(\begin{matrix} f_{M}(a')\left(\frac{\delta}{\left(\delta+\mu+\xi\left(a'\right)\right)\left(\eta_{M}+\mu+\xi\left(a'\right)\right)}\right) \\ f_{S}(a')\left(\frac{\delta}{\left(\delta+\mu+\xi\left(a'\right)\right)\left(\eta_{DS}+\mu+\xi\left(a'\right)\right)}\right) \\ f_{C}(a')\left(\frac{\delta}{\left(\delta+\mu+\xi\left(a'\right)\right)\left(\eta_{DC}+\mu+\xi\left(a'\right)\right)}\right) \end{matrix}\right)$$

whereby $w(a)$ is the proportion of the population in each age group.

**Tables**

**Table S1*.* Definitions of population variables and symbols used in the model.**

| Symbol | Definition |
| --- | --- |
| $S\left(a\right)$ | Susceptible population |
| $E\left(a\right)$ | Latently infected population |
| $I_{M}\left(a\right)$ | Population with mild infection |
| $I_{S}\left(a\right)$ | Population with severe infection |
| $I_{C}\left(a\right)$ | Population with critical infection |
| $D_{S}\left(a\right)$ | Population with severe disease |
| $D_{C}\left(a\right)$ | Population with critical disease |
| $R\left(a\right)$ | Recovered population |
| $Y_{PCR}\left(a\right)$ | Population who test PCR positive after end of infectiousness (prolonged PCR positivity) |
| $X_{IgG}\left(a\right)$ | Population who cleared their infection but have not yet developed detectable antibodies (pre-antibody positivity) |
| $Z_{IgG}\left(a\right)$ | Population who have detectable antibodies (antibody positivity) |
| $N$ | Total population size |
| $n_{age}$ | Number of age groups |
| $\xi(a)$ | Transition rate from one age group to the next age group |
| $\sigma\left(a\right)$ | Age-stratified susceptibility profile to the infection in each age group |
| $1/\delta$ | Duration of latent infection |
| $1/\eta_{M}$ | Duration of mild infection |
| $1/\eta_{DS}$ | Duration of severe infection infectiousness before isolation and/or hospitalization |
| $1/\eta_{S}$ | Duration of severe disease following onset of severe disease |
| $1/\eta_{DC}$ | Duration of critical infection infectiousness before isolation and/or hospitalization |
| $1/\eta_{C}$ | Duration of critical disease following onset of critical disease |
| $1/\vartheta_{PCR}$ | Duration of PCR positivity after end of infectiousness (prolonged PCR positivity) |
| $1/\vartheta_{IgG}$ | Duration between end of infectiousness and development of detectable antibodies (pre-antibody positivity) |
| $1/\mu$ | Natural death rate |
| $\alpha\left(a\right)$ | Disease mortality rate |
| $f_{M}\left(a\right)$ | Proportion of infections that will progress to be mild infections |
| $f_{S}\left(a\right)$ | Proportion of infections that will progress to be severe infections |
| $f_{C}\left(a\right)$ | Proportion of infections that will progress to be critical infections |

**Table S2. Model assumptions in terms of parameter values.**

| **Parameter** | **Symbol** | **Value** | **Justification** |
| --- | --- | --- | --- |
| Duration of latent infection | $1/\delta$ | 3.69 days | Based on existing estimate [4] and based on a median incubation period of 5.1 days [5] adjusted by observed viral load among infected persons [6] and reported transmission before onset of symptoms [7] |
| Duration of infectiousness | $1/\eta_{M}$;$1/\eta_{DS}$; $1/\eta_{DC}$ | 3.48 days | Based on existing estimate[4] and based on observed time to recovery among persons with mild infection [4, 8] and observed viral load in infected persons [6, 7, 9] |
| Duration of severe disease following onset of severe disease | $1/\eta_{S}$ | 28 days | Observed duration from onset of severe disease to recovery [8] |
| Duration of hospitalization for critical infection | $1/\eta_{C}$ | 42 days | Observed duration from onset of critical disease to recovery [8] |
| Life expectancy in Qatar |  | 80.7 years | Estimate for Qatar based on United Nations World Population Prospects database [10] |
| Susceptibility profile to the infection in each age group | $\sigma\left(a\right)$ |  | Model-estimated based on fitting the SARS-CoV-2 epidemic in Qatar [1] |
| Age 0-9 years |  | 0.52 |  |
| Age 10-19 years |  | 0.53 |  |
| Age 20-29 years |  | 0.57 |  |
| Age 30-39 years |  | 0.58 |  |
| Age 40-49 years |  | 0.62 |  |
| Age 50-59 years |  | 0.70 |  |
| Age 60-69 years |  | 0.52 |  |
| Age 70-79 years |  | 0.50 |  |
| Age 80+ years |  | 0.47 |  |
| Proportion of infections that will progress to be infections that require hospitalization in acute care beds | $f_{S}(a)$ |  | The distribution and age dependence of asymptomatic/mild, severe, or critical infections was based on the modeled SARS-CoV-2 epidemic in France [11] |
| Age 0-19 years | $RRS1\times f_{S}$ | $RRS1=0.1$ | Model-estimated relative risk of severe infection based on the SARS-CoV-2 epidemic in France [11] |
| Age 20-29 years | $RRS2\times f_{S}$ | $RRS2=0.5$ | Model-estimated relative risk of severe infection based on the SARS-CoV-2 epidemic in France [11] |
| Age 30-39 years | $f_{S}$ | 0.011 | Model fitting [1] |
| Age 40-49 years | $RRS3\times f_{S}$ | $RRS3=1.2$ | Model-estimated relative risk of severe infection based on the SARS-CoV-2 epidemic in France [11] |
| Age 50-59 years | $RRS4\times f_{S}$ | $RRS4=2.3$ | Model-estimated relative risk of severe infection based on the SARS-CoV-2 epidemic in France [11] |
| Age 60-69 years | $RRS5\times f_{S}$ | $RRS5=4.5$ | Model-estimated relative risk of severe infection based on the SARS-CoV-2 epidemic in France [11] |
| Age 70-79 years | $RRS6\times f_{S}$ | $RRS6=7.8$ | Model-estimated relative risk of severe infection based on the SARS-CoV-2 epidemic in France [11] |
| Age 80+ years | $RRS7\times f_{S}$ | $RRS7=27.6$ | Model-estimated relative risk of severe infection based on the SARS-CoV-2 epidemic in France [11] |
| Proportion of infections that will progress to be infections that require hospitalization in intensive care unit beds | $f_{C}(a)$ |  | The distribution and age dependence of asymptomatic/mild, severe, or critical infections was based on the modeled SARS-CoV-2 epidemic in France [11] |
| Age 0-19 years | $RRC1\times f_{C}$ | $RRC1=0.2$ | Model-estimated relative risk of critical infection based on the SARS-CoV-2 epidemic in France [11] |
| Age 20-29 years | $RRC2\times f_{C}$ | $RRC2=0.3$ | Model-estimated relative risk of critical infection based on the SARS-CoV-2 epidemic in France [11] |
| Age 30-39 years | $f_{C}$ | 0.002 | Model fitting [1] |
| Age 40-49 years | $RRC3\times f_{C}$ | $RRC3=1.8$ | Model-estimated relative risk of critical infection based on the SARS-CoV-2 epidemic in France [11] |
| Age 50-59 years | $RRC4\times f_{C}$ | $RRC4=4.7$ | Model-estimated relative risk of critical infection based on the SARS-CoV-2 epidemic in France [11] |
| Age 60-69 years | $RRC5\times f_{C}$ | $RRC5=10.6$ | Model-estimated relative risk of critical infection based on the SARS-CoV-2 epidemic in France [11] |
| Age 70-79 years | $RRC6\times f_{C}$ | $RRC6=13.6$ | Model-estimated relative risk of critical infection based on the SARS-CoV-2 epidemic in France [11] |
| Age 80+ years | $RRC7\times f_{C}$ | $RRC7=8.7$ | Model-estimated relative risk of critical infection based on the SARS-CoV-2 epidemic in France [11] |

**Additional Figures**

**Figure S1: Schematic diagram presenting the basic structure of the mathematical model for SARS-CoV-2 transmission dynamics with the prolonged PCR positivity and delayed antibody detection**.

**
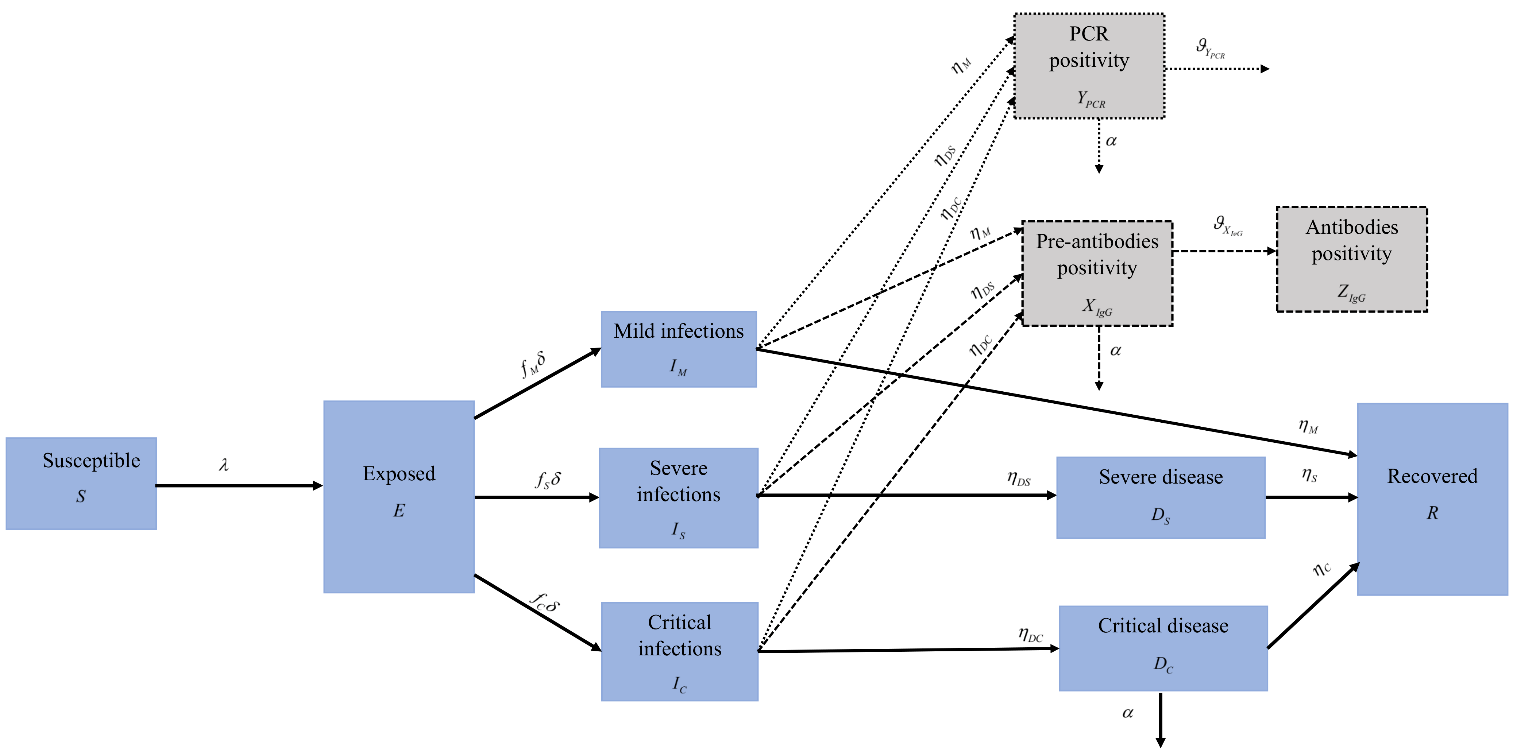
**

**Figure S2. Effect of the prolonged PCR positivity on the observed SARS-CoV-2 positivity rate.** Ratio of the proportion of tests that are PCR positive (“positivity rate”) in presence of the prolonged PCR positivity over the proportion of tests that are PCR positive assuming no prolonged PCR positivity. The prolonged PCR positivity is assumed to last on average for 2, 3, 4, and 6 weeks. In this epidemic simulation, *R*_0_ has a value of 3.0, that is the natural course of the epidemic in absence of any social or physical distancing interventions.


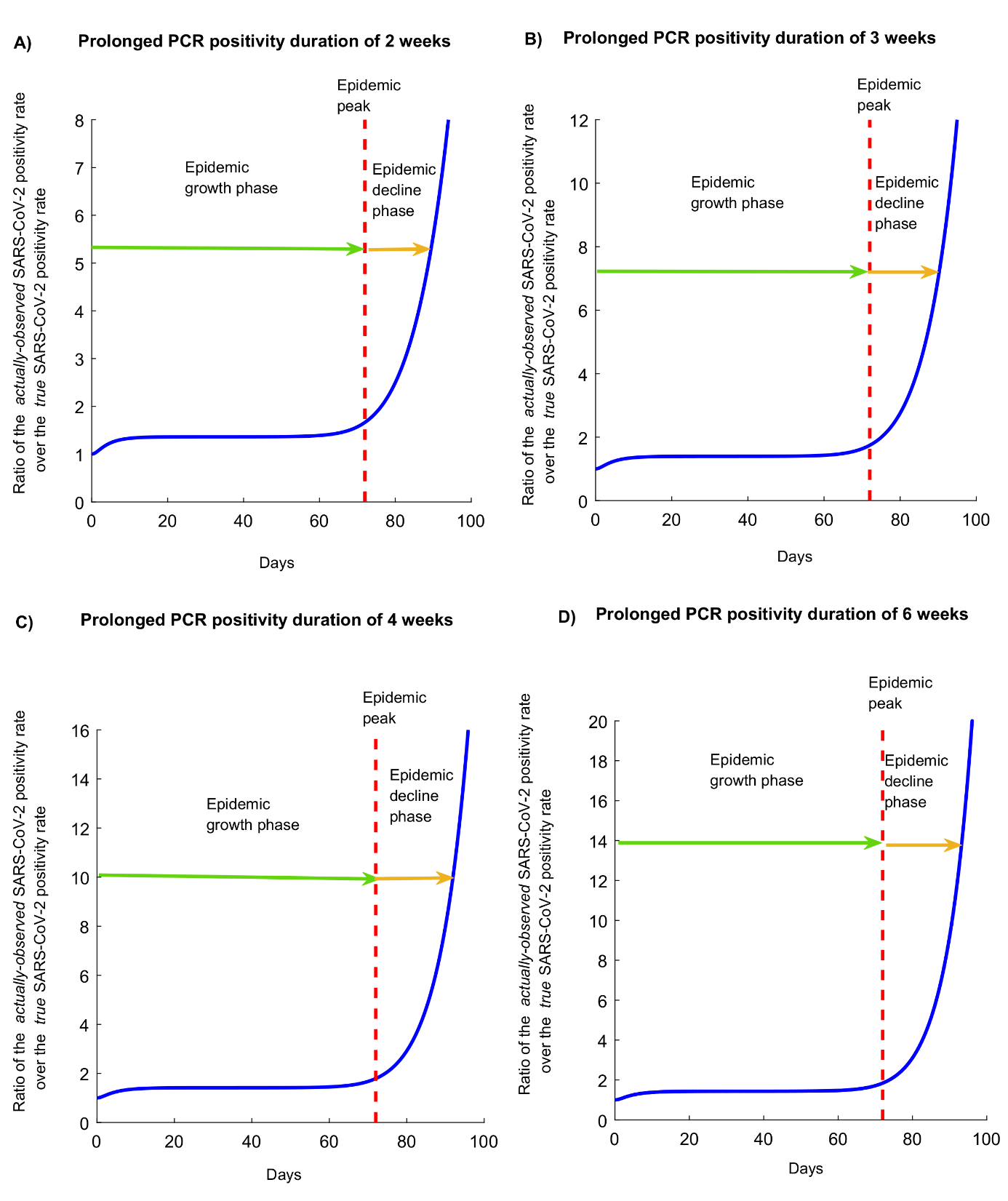


**Figure S3. Effect of the prolonged PCR positivity on the observed distribution of those who are latently infected, infectious, and post-infectious.** Proportion of new diagnoses who are in latent infection, stage of infectiousness, or stage of prolonged PCR positivity. The prolonged PCR positivity is assumed to last on average for three weeks after end of infectiousness [12, 13]. In this epidemic simulation, *R*_0_ has a value of 3.0, that is the natural course of the epidemic in absence of any social or physical distancing interventions.

**
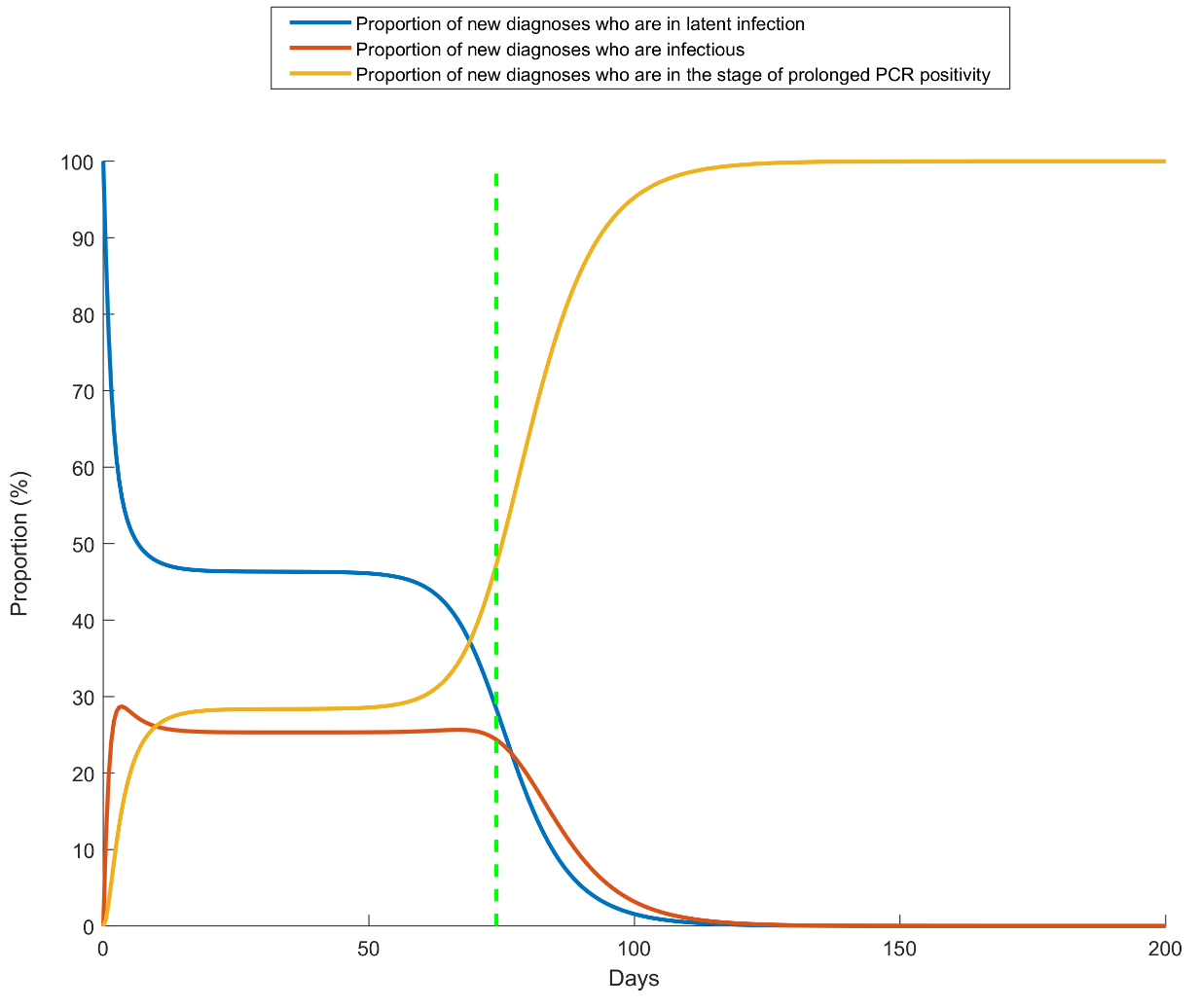
**

**Figure S4. Effect of the prolonged PCR positivity on estimation of the basic reproduction number *R_0_* using the *actually-observed* trend in diagnosed cases.** The estimated *R*_0_ as derived from the epidemic curve of diagnosed cases *in presence* and *in absence* of the prolonged PCR positivity. The prolonged PCR positivity is assumed to last on average for 2, 3, 4, and 6 weeks. Two scenarios are presented, one for an *R*_0_ of 1.6 (an epidemic in presence of social and physical distancing interventions) and an *R*_0_ of 3.0 (natural course of the epidemic in absence of any social or physical distancing interventions).


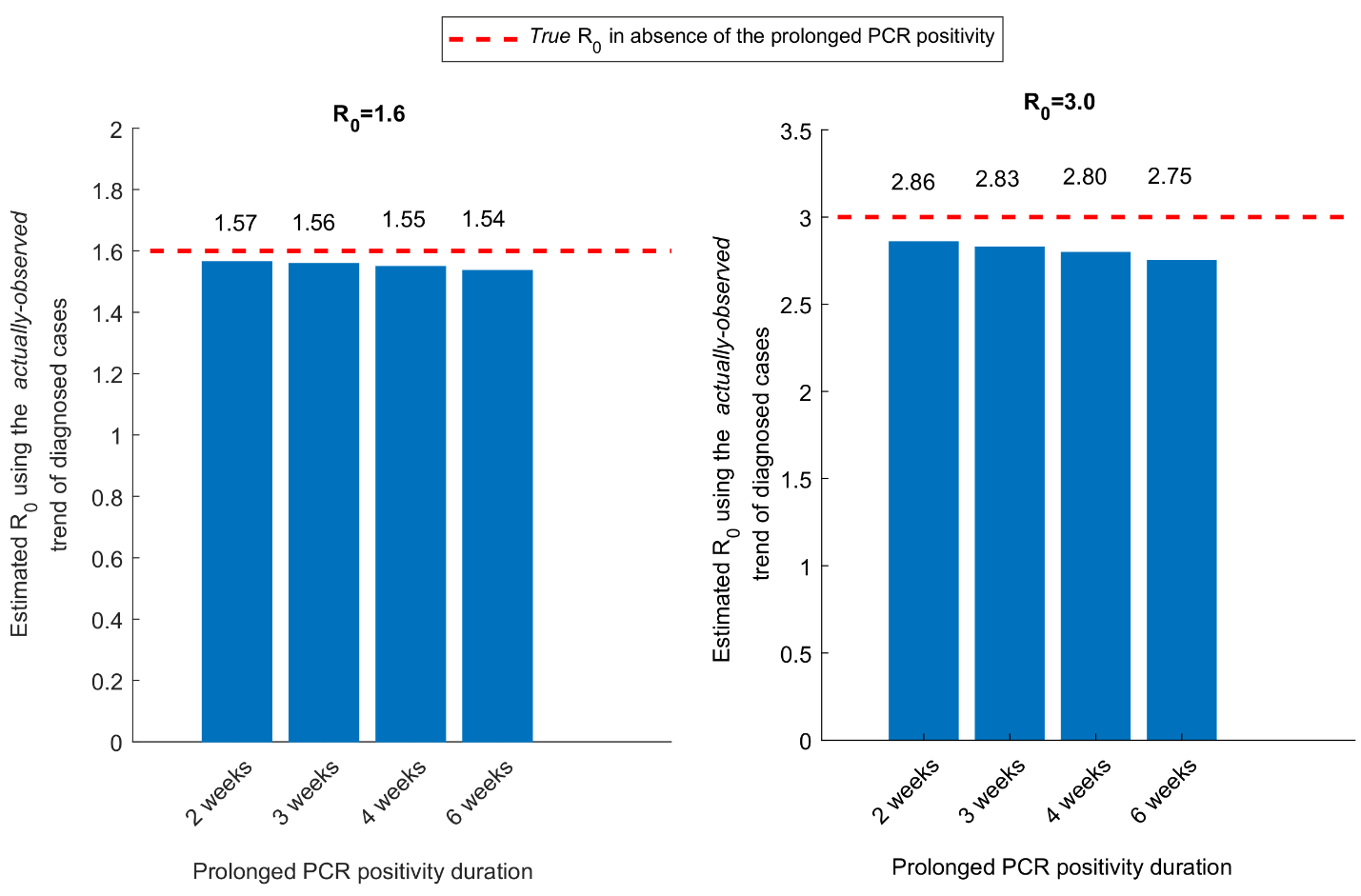


**References**

1. Abu-Raddad LJ, Chemaitelly H, Ayoub HH, Al Kanaani Z, Al Khal A, Al Kuwari E, Butt AA, Coyle P, Jeremijenko A, Kaleeckal AH *et al*: **Characterizing the Qatar advanced-phase SARS-CoV-2 epidemic**. *medRxiv* 2020:2020.2007.2016.20155317v20155312 (non-peer-reviewed preprint).

2. Ayoub HH, Chemaitelly H, Seedat S, Makhoul M, Al Kanaani Z, Al Khal A, Al Kuwari E, Butt AA, Coyle P, Jeremijenko A *et al*: **Mathematical modeling of the SARS-CoV-2 epidemic in Qatar and its impact on the national response to COVID-19**. *medRxiv* 2020:2020.2011.2008.20184663 (non-peer-reviewed preprint).

3. Heffernan JM, Smith RJ, Wahl LM: **Perspectives on the basic reproductive ratio**. *Journal of the Royal Society Interface* 2005, **2**(4):281-293.

4. Li R, Pei S, Chen B, Song Y, Zhang T, Yang W, Shaman J: **Substantial undocumented infection facilitates the rapid dissemination of novel coronavirus (SARS-CoV2)**. *Science* 2020, **368**(6490):489-493.

5. Lauer SA, Grantz KH, Bi Q, Jones FK, Zheng Q, Meredith HR, Azman AS, Reich NG, Lessler J: **The Incubation Period of Coronavirus Disease 2019 (COVID-19) From Publicly Reported Confirmed Cases: Estimation and Application**. *Ann Intern Med* 2020, **172**(9):577-582.

6. Zou L, Ruan F, Huang M, Liang L, Huang H, Hong Z, Yu J, Kang M, Song Y, Xia J *et al*: **SARS-CoV-2 Viral Load in Upper Respiratory Specimens of Infected Patients**. *N Engl J Med* 2020, **382**(12):1177-1179.

7. Rothe C, Schunk M, Sothmann P, Bretzel G, Froeschl G, Wallrauch C, Zimmer T, Thiel V, Janke C, Guggemos W *et al*: **Transmission of 2019-nCoV Infection from an Asymptomatic Contact in Germany**. *N Engl J Med* 2020, **382**(10):970-971.

8. World Health Organization: **Report of the WHO-China Joint Mission on Coronavirus Disease 2019 (COVID-19). Available from :**[**https://www.who.int/docs/default-source/coronaviruse/who-china-joint-mission-on-covid-19-final-report.pdf**](https://www.who.int/docs/default-source/coronaviruse/who-china-joint-mission-on-covid-19-final-report.pdf)**. Accessed on March 10, 2020**. In*.*; 2020.

9. He X, Lau EHY, Wu P, Deng X, Wang J, Hao X, Lau YC, Wong JY, Guan Y, Tan X *et al*: **Temporal dynamics in viral shedding and transmissibility of COVID-19**. *Nat Med* 2020, **26**(5):672-675.

10. United Nations Department of Economic and Social Affairs Population Dynamics: **The 2019 Revision of World Population Prospects. Available from** [**https://population.un.org/wpp/**](https://population.un.org/wpp/)**. Accessed on March 1st, 2020.** 2020.

11. Salje H, Tran Kiem C, Lefrancq N, Courtejoie N, Bosetti P, Paireau J, Andronico A, Hoze N, Richet J, Dubost CL *et al*: **Estimating the burden of SARS-CoV-2 in France**. *Science* 2020:eabc3517.

12. Sethuraman N, Jeremiah SS, Ryo A: **Interpreting Diagnostic Tests for SARS-CoV-2**. *JAMA* 2020.

13. Wajnberg A, Mansour M, Leven E, Bouvier NM, Patel G, Firpo-Betancourt A, Mendu R, Jhang J, Arinsburg S, Gitman M *et al*: **Humoral response and PCR positivity in patients with COVID-19 in the New York City region, USA: an observational study**. *Lancet Microbe* 2020, **1**(7):e283-e289.
